# SurvDB: Systematic Identification of Potential Prognostic Biomarkers in 33 Cancer Types

**DOI:** 10.3390/ijms26062806

**Published:** 2025-03-20

**Authors:** Zejun Wu, Congcong Min, Wen Cao, Feiyang Xue, Xiaohong Wu, Yanbo Yang, Jianye Yang, Xiaohui Niu, Jing Gong

**Affiliations:** 1 Hubei Key Laboratory of Agricultural Bioinformatics, College of Informatics, Huazhong Agricultural University, Wuhan 430074, China; wuzejun@webmail.hzau.edu.cn (Z.W.); mincongcong@webmail.hzau.edu.cn (C.M.); niuxiaoh@mail.hzau.edu.cn (X.N.); 2 College of Biomedicine and Health, Huazhong Agricultural University, Wuhan 430070, China

**Keywords:** cancer prognosis, survival analysis, biomarkers identification

## Abstract

The identification of cancer prognostic biomarkers is crucial for predicting disease progression, optimizing personalized therapies, and improving patient survival. Molecular biomarkers are increasingly being identified for cancer prognosis estimation. However, existing studies and databases often focus on single-type molecular biomarkers, deficient in comprehensive multi-omics data integration, which constrains the comprehensive exploration of biomarkers and underlying mechanisms. To fill this gap, we conducted a systematic prognostic analysis using over 10,000 samples across 33 cancer types from The Cancer Genome Atlas (TCGA). Our study integrated nine types of molecular biomarker-related data: single-nucleotide polymorphism (SNP), copy number variation (CNV), alternative splicing (AS), alternative polyadenylation (APA), coding gene expression, DNA methylation, lncRNA expression, miRNA expression, and protein expression. Using log-rank tests, univariate Cox regression (uni-Cox), and multivariate Cox regression (multi-Cox), we evaluated potential biomarkers associated with four clinical outcome endpoints: overall survival (OS), disease-specific survival (DSS), disease-free interval (DFI), and progression-free interval (PFI). As a result, we identified 4,498,523 molecular biomarkers significantly associated with cancer prognosis. Finally, we developed SurvDB, an interactive online database for data retrieval, visualization, and download, providing a comprehensive resource for biomarker discovery and precision oncology research.

## 1. Introduction

Cancer is a leading cause of global mortality [1]. According to the World Health Organization (WHO), approximately 20 million new cases and 9.7 million deaths reported worldwide in 2022 [2]. Precision therapy based on cancer prognostic biomarkers can significantly extend survival and improve the quality of life for cancer patients [3].

Cancer prognosis refers to the estimation of patient endpoints, including survival duration, recurrence risk, or disease progression after diagnosis [4]. Clinical outcome endpoints include overall survival (OS), disease-specific survival (DSS), disease-free interval (DFI), and progression-free interval (PFI) [5]. OS measures the time from diagnosis or treatment to death from any cause, providing a general survival assessment. DSS measures the time from diagnosis or treatment to death specifically caused by the disease, providing a clearer assessment of disease-specific lethality. DFI measures the time from complete remission (e.g., no residual tumor after surgery) to disease recurrence, assessing the duration of a disease-free state. PFI measures the time from stable disease to biological progression. While OS and DSS focus on survival duration, DFI and PFI emphasize the evaluation of disease status, as DFI highlights recurrence after a disease-free state and PFI tracks disease progression under controlled conditions, making them more critical for assessing the underlying dynamics of disease than OS and DSS [6,7]. In precision medicine, DFI and PFI have gained increasing attention as indicators of treatment efficacy, as they reflect the effects of therapy on disease control, enabling clinicians to promptly adjust treatment plans and achieve the goals of precision medicine. Moreover, given the relatively short clinical follow-up records for most of The Cancer Genome Atlas (TCGA) cohorts, PFI and DFI might generally be considered better endpoints choices than OS and DSS [8]. However, to our knowledge, current databases lack sufficient focus on PFI and DFI.

Previous studies have revealed significant variations in survival across cancer types. For instance, thyroid carcinoma (THCA) exhibits a relatively high five-year survival rate of 92.9%, in contrast to pancreatic adenocarcinoma (PAAD), which has a markedly lower rate of 8.5% [9]. Even within the same cancer type, OS varies considerably. In TCGA database, the median OS for skin cutaneous melanoma (SKCM) is 36.4 months, yet 14.7% of cases survive over 10 years. These results show the high heterogeneity of cancer prognosis. Investigating factors influencing cancer prognosis is essential for understanding cancer progression mechanisms and can inform clinical decision making and treatment efficacy evaluation [8,10]. For instance, in HER2-positive breast cancer, trastuzumab-based combination therapy significantly improves patient survival and quality of life [11,12].

With advancements in sequencing technologies and the increasing demands of precision medicine, cancer prognosis biomarker research has shifted from traditional clinical and demographic indicators to molecular level precision and personalized biomarker assessments [13,14]. Numerous omics data, including genomics, transcriptomics, and proteomics, are increasingly used for prognostic analysis [15,16,17]. Various biomarkers from different omics layers, such as single-nucleotide polymorphism (SNP), DNA methylation, and gene expression level, have been associated with cancer prognosis. For instance, the SNP rs27770A > G variant in the *PLK1* has been reported to reduce its binding affinity to miRNA, suppressing mRNA expression and influencing liver hepatocellular carcinoma (LIHC) prognosis [18]. Biomarkers such as *TP53* [19] and *PD-L1* [20] have also been linked to multiple cancer prognoses and demonstrate promising potential in clinical applications. Copy number variations (CNVs) have been used to estimate some cancer progression [21,22]. Long non-coding RNA (lncRNA) is used to evaluate the diagnosis and treatment of non-triple-negative and triple-negative breast cancer and other cancer [23,24]. Alternative polyadenylation (APA) might lead to a worse prognosis in some cancers [25]. In cancer, aberrant alternative splicing (AS) patterns are frequently observed and known to contribute to carcinogenesis, de-differentiation, and metastasis [26]. Aberrant DNA methylation has been observed in various human diseases, including cancer [27]. However, to our knowledge, existing studies and databases are often limited to single-type molecular biomarkers for cancer prognosis.

TCGA database provides clinical and multi-omics data, including genomic, transcriptomic, proteomic, and methylation data, for over 10,000 samples across 33 cancer types, offering a valuable resource for cancer prognosis research. Several prognostic databases have been developed based on TCGA, such as GEPIA2 [28], TCPA [29], SurvivalMeth [30], OncoSplicing [31], and OSppc [32]. However, these databases primarily focus on single-type molecular biomarkers and lack comprehensive integration and analysis capabilities for multiple biomarker types, limiting the exploration of the complex molecular mechanisms underlying cancer prognosis. Additionally, some types of molecular data like genomic variant, CNV, APA, AS, and miRNA expression remain underexplored in prognostic studies. Furthermore, many databases assess only 1–2 clinical outcomes, such as OS or DSS, while neglecting DFI and PFI. To address these limitations, we systematically analyzed the relationship between nine types of molecular data and four clinical outcomes (OS, DSS, PFI, DFI). Additionally, we developed a comprehensive cancer prognosis database SurvDB (https://gong_lab.hzau.edu.cn/SurvDB/, accessed on 1 January 2025), an interactive online database for data retrieval, visualization, and download. SurvDB provides a comprehensive resource for candidate biomarker discovery and precision oncology research, and will support the exploration of complex molecular mechanisms underlying cancer prognosis.

## 2. Results

### 2.1. Data Summary of SurvDB

In SurvDB, we used multi-omics and clinical data from 33 cancer types available in TCGA database, encompassing 11,160 tumor samples. The sample size for each cancer type ranged from 12 in uveal melanoma (UVM) to 1207 in breast invasive carcinoma (BRCA) (Table 1).

From TCGA and its derivative databases, we integrated nine types of molecular biomarker-related data: SNP, CNV, AS, APA, coding gene expression, DNA methylation, lncRNA expression, miRNA expression, and protein expression. To ensure consistency and simplify subsequent descriptions, all molecules or indices within these datasets are collectively referred to as “markers”. In total, we analyzed the relationship between 6,867,129 markers and cancer prognosis (Table 2). Of them, the SNP, CNV, mRNA expression, lncRNA expression, miRNA expression and DNA methylation data were directly downloaded from TCGA database, while the Percentage of Distal polyA site Usage Index (PDUI) data for APA events, percent spliced index (PSI) data for AS events, and protein expression data were downloaded from TC3A database, TCGA SpliceSeq, and TCPA database, respectively [29,33,34].

After filtering by imputation score, minor allele frequency (MAF), missing rate, and Hardy–Weinberg *p*-value, an average of 3,352,031 SNPs per cancer type were retained. For mRNA, lncRNA, and miRNA, after removing low-expression genes (FPKM < 0.01 for mRNA and lncRNA, TPM < 0.01 for miRNA), an average of 16,894 coding genes, 7634 lncRNAs, and 480 miRNAs were retained per cancer type. After quality control, an average of 3846 APA events, 25,937 alternative splicing (AS) events, 366,644 DNA methylation sites, 19,629 CNVs and 219 proteins per cancer type were retained for downstream analysis.

Potential prognostic biomarkers for nine types of molecular data across 33 cancers were identified through Log-rank test, uni-Cox, and multi-Cox, with *p* < 0.05 as the significance threshold. A total of 4,498,523 unique prognostic biomarkers were identified across four clinical outcomes, encompassing nine types of molecular data. (Table 3), including 4,035,082 SNPs, 17,730 coding genes, 9837 lncRNAs, 851 miRNAs, 446 proteins, 367,478 methylation sites, 35,695 AS events, 6942 APA events, and 24,462 CNVs.

### 2.2. Database Construction

All results were stored in a MongoDB database (v3.4.2). A user-friendly web interface, SurvDB (https://gong_lab.hzau.edu.cn/SurvDB/, accessed on 1 January 2025), was developed using the Flask framework (v1.0.3) to support data browsing, searching, and downloading. The database operates on an Apache2 web server (v2.4.18) and is compatible with multiple browsers across various operating systems.

### 2.3. Functions and Usage of SurvDB

**Figure 1:**
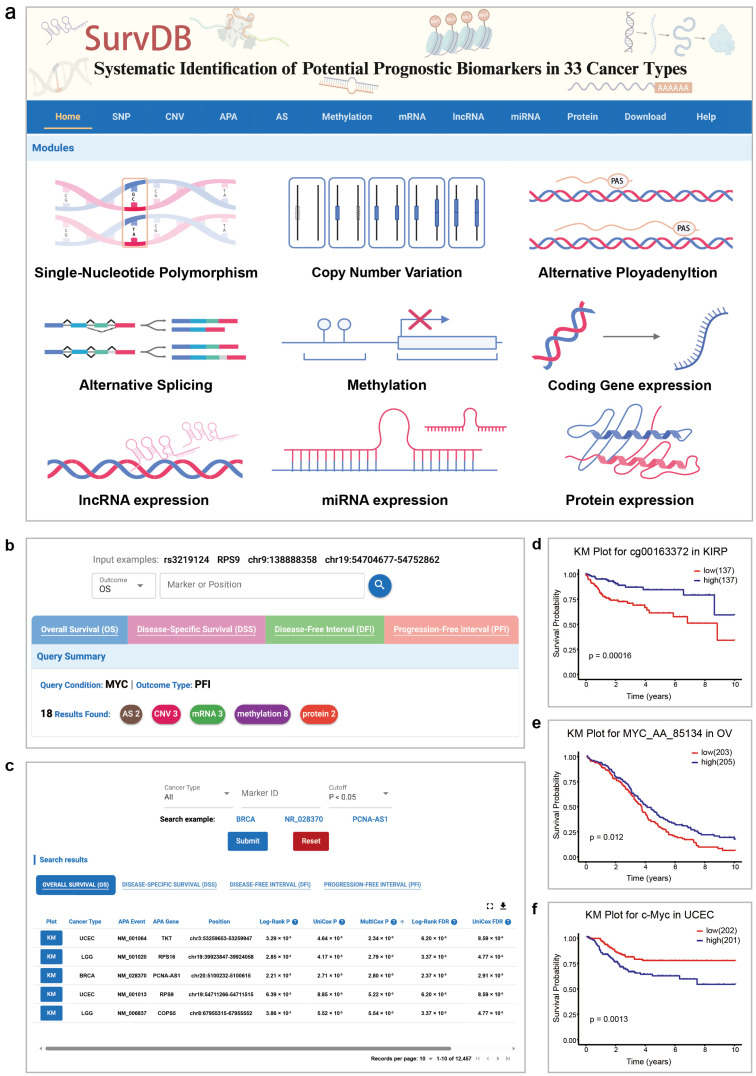
The interface of SurvDB. (**a**) Browser bar in SurvDB and main modules in SurvDB, including SNP, CNV, APA, AS, Methylation, mRNA, lncRNA, miRNA, protein and download modules. (**b**) Results of *MYC* gene by aggregation query on the home page. (**c**) The query page for a specific type of molecular marker. (**d**–**f**) Examples of methylation, AS, and protein results in *MYC* search results.

SurvDB provides a user-friendly web interface for users to browse, visualize, search, and download prognostic biomarkers of different types (Figure 1). To fully utilize multi-omics data, we designed an aggregation query module. By entering a marker name or a genomic region, users can obtain integrated search results, including all related information across multiple cancers or types of molecular markers. For example, multiple genetic loci, APA, and CNV in the chromosomal region 9q21.3 (chr9:21800000-22400000) have been reported associated with various cancers [35]. Users can input chr9:21800000-22400000 to retrieve all potential prognostic biomarkers identified across different types of molecular data in that region. Additionally, multiple types of molecular markers are linked with genes, and the results for these genes across four clinical outcomes are shown. By entering a specific gene, users can obtain related molecular markers associated with four clinical outcomes. For example, for the *MYC* gene, 15 results are shown in OS section. As for other three outcomes (DSS, DFI, PFI), there are 17, 11, and 18 results. In PFI section, the 18 results include 8 methylation records, 3 records each for mRNA and CNV, and 2 records each for AS and protein (Figure 1b).

On the separate query page for each type of molecular data (Figure 1c), users can query analysis results for individual markers based on cancer type, marker ID, and genomic location. The Kaplan–Meier (KM) plotter shows examples of methylation, AS, and protein results in *MYC* search results, respectively (Figure 1d–f). The “Help” page offers database descriptions and usage guides, and feedback can be emailed using the address at the page’s bottom.

## 3. Discussion

This study utilized multi-omics data provided by TCGA database and systematically analyzed nine types of molecular data to identify potential prognostic biomarkers by combining three classic survival analysis methods: Log-rank test, uni-Cox, and multi-Cox. Additionally, to better present the results, we developed a user-friendly SurvDB database to facilitate querying, browsing, and downloading by users.

Compared to other cancer prognosis-related databases, SurvDB offers the following advantages. First, survdb incorporates more types of molecular markers, such as genomic variations, CNV, APA, and miRNA expression. Research has shown that APA of the *CSTF2* is associated with lung cancer prognosis [36], and *miRNA-21* is related to the prognosis of metastatic colorectal cancer [37]. A systematic analysis of these types of molecular markers and their relationship with cancer prognosis will provide more insights for further biological experiments and clinical research.

The integration of multi-omics data offers a new perspective for cancer prognosis research. By combining various omics data, such as gene expression, mutation, and epigenetic information, it is possible to gain a more comprehensive understanding of the complexity of cancer and improve the accuracy of prognosis prediction. For instance, a study based on TCGA constructed a lung adenocarcinoma prognosis-related risk prediction model by integrating multi-omics data, demonstrating the potential of multi-omics data in improving prognosis accuracy [38]. Additionally, a study on liver cancer, published by the collaborative team of Tsinghua University, also highlighted the value of multi-omics data in cancer prognosis research [39]. The prognostic findings of multiple types of molecular markers in this study offer insights for multi-omics feature selection.

This study also identified different types of molecular biomarkers associated with DFI and PFI, addressing the lack of focus on clinical outcome endpoints in other databases. These findings contribute to the mechanistic exploration of DFI and PFI. SurvDB also features a multi-cancer and multi-molecule joint query function, which helps other researchers conduct multi-level mechanistic investigations.

The integrative identification of prognostic biomarkers enhances understanding of cancer progression and aids in identifying high-risk patients, offering valuable guidance for clinical decision making. Cancer recurrence is a specific prognostic outcome. For example, Professor Luo’s team from Sun Yat-sen University [40] combined TCGA renal cancer data with data from 227 Chinese patients and used the multicenter retrospective analysis method to identify six SNPs closely associated with localized renal cancer recurrence in Chinese populations. They further demonstrated that integrating these six SNPs into a predictive model alongside clinical pathological indicators improved prediction accuracy, enabling the more precise identification of high-risk patients for recurrence. They proposed that intensified monitoring and adjuvant therapy for high-risk patients could mitigate adverse outcomes.

In the future, we will explore artificial intelligence and machine learning techniques to fully utilize the identified biomarkers for constructing multi-omics predictive models, aiming to improve the accuracy of prognosis prediction and its clinical applicability. Additionally, this research has revealed significant prognostic variability across populations, while TCGA data mainly represent Western cohorts [41]. Thus, we aim to expand the SurvDB database by incorporating data from diverse populations, additional cancer subtypes, and broader biomarker categories. Ultimately, we hope that SurvDB will become a vital resource for cancer prognosis researchers, promoting advancements in cancer prognosis studies and precision medicine.

## 4. Materials and Methods

### 4.1. Molecular Data Collection and Processing

**Figure 2:**
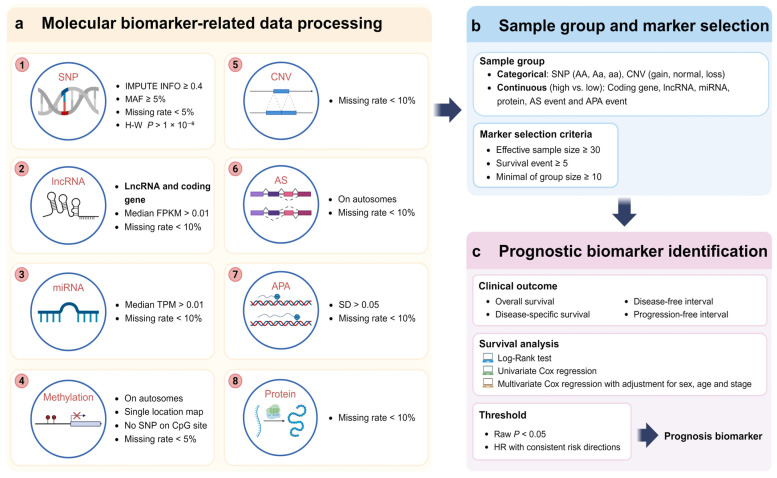
The pipeline of SurvDB. (**a**) Processing of each molecular biomarker-related data. (**b**) Sample grouping and marker filtering. (**c**) Identification of prognostic biomarkers. Three survival analysis methods were used to evaluate the associations between markers and four clinical outcomes.

Nine types of molecular biomarker-related data were collected from TCGA and its derivative databases, followed by processing and quality control (Figure 2). Genotype data detected using the Affymetrix SNP 6.0 array were obtained from TCGA. We imputed autosomal variants in all samples for each cancer type using IMPUTE2 (v2.3.2), with the 1000 Genomes Phase 3 as the reference panel [42]. The imputation was performed in the two-step procedure provided by IMPUTE2. After imputation, SNPs were filtered using the following criteria: (i) imputation quality score ≥ 0.4, (ii) minor allele frequency (MAF) ≥ 5%, (iii) missing rate < 5%, and (iv) Hardy–Weinberg equilibrium *p*-value > 1 × 10^−6^.

For the quality control of coding gene and lncRNA, we downloaded the gene expression profile from TCGA and excluded all the genes with an extremely low expression median fragment per kilobase million (FPKM) < 0.01 and a missing rate > 10% for downstream analysis. Then, genes were classified into coding gene and lncRNA according to the annotation from ENCODE (v36) [26,27].

For the quality control of miRNA, we downloaded the miRNA sequencing data from TCGA and excluded the miRNAs with a median transcription per million (TPM) < 0.01 and those with missing rate > 10%.

PSI, a commonly used metric for quantifying splicing events, is defined as the ratio of reads indicating the presence of a transcript element versus the total number of reads covering the splicing event. After downloading PSI data from TCGA SpliceSeq [34], we excluded AS events with a missing rate > 10% across all samples or those located on sex chromosomes.

PDUI quantifies the alternative polyadenylation (APA) frequency based on the relative usage of distal polyA sites. The PDUI data were obtained from TC3A database [33]. APA events with a missing rate >10% or a standard deviation < 5% were excluded.

DNA methylation data were obtained from TCGA database, generated using the Illumina Infinium HumanMethylation450 BeadChip array. We downloaded these data from TCGA data portal and filtered out the sites according to the following criteria: (i) on sex chromosomes; (ii) mapping to multiple locations on the genome; (iii) containing known SNP on CpG sites; and (iv) beta value with a missing rate > 5%.

CNV data were retrieved from TCGA and further processed using GISTIC2.0 to discretize CNV states into five categories: homozygous deletion (−2), single-copy loss (−1), diploid (0), low-level gain (1), and high-level amplification (2) [43]. CNV data were mapped to human genome coordinates using the HUGO probeMap from UCSC Xena to determine the copy number state for each gene [44].

Protein expression data were derived from reverse-phase protein array (RPPA) experiments available in TCGA. Expression levels for 282 proteins were obtained from TCPA database and further annotated at gene level.

### 4.2. Clinical Data Collection and Processing

Clinical data were obtained from TCGA database, including patient age, sex, tumor stage, OS, DSS, DFI, and PFI. Redundant samples from the same patient were excluded. For each cancer type, only patient samples with complete clinical information were included in the analysis.

### 4.3. Identification of Prognostic Biomarkers

During the identification of genotype- and CNV-related prognostic biomarkers, samples were grouped based on their classification. To ensure the reliability of subsequent survival analysis results, markers with fewer than 30 valid samples or fewer than 5 samples in the smallest group were excluded from the analysis. For other types of molecular markers, in each analysis, samples were grouped into two groups based on the median value of the marker. Next, we employed three classic survival analysis methods, Log-rank test, uni-Cox, and multi-Cox, to systematically assess the correlation between markers and cancer patient outcomes, including OS, DSS, PFI, and DFI. The multi-Cox model further incorporated covariates such as gender, diagnosis age, and tumor stage to adjust for potential confounders.

As a database, we aim to retain more information on significant finding. Therefore, markers that were consistently significant (raw *p*-value < 0.05) and exhibited consistent risk directions across all methods were selected as possible prognostic biomarkers.

## Figures and Tables

**Table 1:** Summary of sample sizes for types of molecular data in SurvDB.

Cancer	APA Event	AS Event	CNV	SNP	Total RNA	miRNA	Methylation	Protein
Adrenocortical carcinoma (ACC)	79	79	90	77	79	79	80	46
Bladder urothelial carcinoma (BLCA)	408	425	408	408	406	409	412	344
Breast invasive carcinoma (BRCA)	1095	1207	1080	1092	1095	750	792	887
Cervical squamous cell carcinoma and endocervical adenocarcinoma (CESC)	304	256	295	300	304	306	307	173
Cholangiocarcinoma (CHOL)	36	45	36	36	36	36	36	30
Colon adenocarcinoma (COAD)	624	499	451	286	456	259	297	360
Lymphoid neoplasm diffuse large B-cell lymphoma (DLBC)	48	48	48	48	48	47	48	33
Esophageal carcinoma (ESCA)	184	193	184	184	165	182	185	126
Glioblastoma Multiforme (GBM)	161	160	577	150	166	-	142	238
Head and neck squamous cell carcinoma (HNSC)	520	544	522	518	503	484	528	357
Kidney chromophobe (KICH)	66	91	66	66	66	65	66	63
Kidney renal clear cell carcinoma (KIRC)	531	605	528	527	532	243	319	478
Kidney renal papillary cell carcinoma (KIRP)	290	322	288	290	290	286	275	215
Acute myeloid leukemia (LAML)	172	178	191	123	150	188	194	-
Lower grade glioma (LGG)	516	515	513	515	514	510	516	430
Liver hepatocellular carcinoma (LIHC)	371	421	370	369	371	370	377	184
Lung adenocarcinoma (LUAD)	512	573	516	514	517	454	461	365
Lung squamous cell carcinoma (LUSC)	501	550	501	500	501	336	374	328
Mesothelioma (MESO)	87	87	87	87	87	87	87	63
Ovarian serous cystadenocarcinoma (OV)	412	420	579	301	378	477	10	426
Pancreatic adenocarcinoma (PAAD)	178	182	184	178	178	177	184	123
Pheochromocytoma and paraganglioma (PCPG)	179	181	162	178	179	178	179	80
Prostate adenocarcinoma (PRAD)	497	549	492	494	497	491	498	352
Rectum adenocarcinoma (READ)	-	176	165	94	167	92	98	131
Sarcoma (SARC)	259	261	257	258	259	256	261	223
Skin cutaneous melanoma (SKCM)	469	104	367	103	469	448	471	352
Stomach adenocarcinoma (STAD)	415	452	441	415	380	387	396	357
Testicular germ cell tumors (TGCT)	150	149	150	150	150	149	150	118
Thyroid carcinoma (THCA)	505	564	499	503	504	502	507	372
Thymoma (THYM)	120	122	123	120	120	124	124	90
Uterine corpus endometrial carcinoma (UCEC)	545	580	539	176	557	411	444	440
Uterine carcinosarcoma (UCS)	57	57	56	56	57	56	57	48
Uveal melanoma (UVM)	80	80	80	80	80	80	80	12

**Table 2:** Numbers of retained markers for types of molecular data in SurvDB.

Cancer	APA Event	AS Event	CNV	SNP	mRNA	lncRNA	miRNA	Methylation	Protein
ACC	3008	20,087	21,518	2,400,199	16,347	6418	486	367,935	220
BLCA	3522	24,413	24,728	3,802,497	16,794	7235	471	367,266	216
BRCA	4974	29,617	24,777	2,690,254	16,991	7847	441	366,485	217
CESC	2999	25,883	24,541	3,666,670	16,781	7228	484	365,963	219
CHOL	3421	22,868	8357	1,704,318	16,705	7127	474	358,897	218
COAD	3193	20,305	24,520	3,878,474	16,655	6383	473	366,918	223
DLBC	3274	19,761	5254	2,592,106	16,380	6663	469	366,641	218
ESCA	4133	35,860	24,520	3,571,957	17,571	9753	460	365,560	219
GBM	5144	30,304	24,015	3,168,354	17,293	8565	-	367,156	223
HNSC	4399	27,375	24,756	3,993,242	16,838	6864	500	367,308	217
KICH	4250	29,112	9708	2,164,115	16,602	7394	447	367,363	219
KIRC	4614	30,673	24,360	4,216,575	17,019	8565	401	367,435	233
KIRP	3806	25,207	18,452	4,021,930	16,763	7542	432	367,054	220
LAML	2983	21,157	4230	3,253,971	16,852	8881	330	367,832	-
LGG	4868	32,685	24,085	4,233,472	17,154	8971	514	367,493	217
LIHC	2852	19,398	24,287	3,601,198	16,115	5877	472	366,485	219
LUAD	4285	28,262	24,777	4,003,422	17,103	8002	476	367,041	216
LUSC	4910	30,908	24,436	3,470,575	17,288	8330	501	367,396	216
MESO	3735	27,337	14,401	3,054,234	16,834	7453	496	367,664	219
OV	5834	31,830	24,776	2,670,787	17,331	8838	440	-	224
PAAD	4142	29,057	24,279	3,980,971	17,286	8190	506	364,850	218
PCPG	3434	23,801	13,476	3,362,598	16,679	7405	534	367,579	219
PRAD	4271	27,776	23,413	4,225,082	17,055	7737	416	367,372	217
READ	-	20,544	22,639	2,844,658	16,705	6451	500	366,534	223
SARC	3528	24,440	24,748	3,499,520	16,622	7051	388	364,539	219
SKCM	4231	25,492	24,504	3,122,533	16,536	6901	491	366,340	216
STAD	6138	32,343	24,538	3,878,917	17,520	9402	443	365,707	217
TGCT	4321	26,158	19,896	3,405,712	17,739	8759	679	367,904	216
THCA	477	28,762	10,043	4,262,697	16,716	7595	489	367,735	217
THYM	3344	22,009	4674	3,229,257	17,010	7983	617	367,897	218
UCEC	2480	15,266	24,453	3,679,658	17,035	6906	504	367,681	223
UCS	3491	24,182	20,361	2,075,347	17,376	8137	528	364,672	219
UVM	3007	23,033	10,223	2,891,714	15,819	5477	494	367,920	-

**Table 3:** Numbers of identified potential prognostic biomarkers for types of molecular data in SurvDB.

Cancer	APA Event	AS Event	CNV	SNP	mRNA	lncRNA	miRNA	Methylation	Protein
ACC	250	2945	6197	132,023	4522	899	156	82,413	26
BLCA	989	4276	3025	313,569	3154	1339	81	56,240	44
BRCA	1051	5089	3026	255,566	3578	1155	174	85,085	77
CESC	685	4197	2293	343,955	3619	916	159	80,185	47
CHOL	288	1759	339	131,598	1430	397	47	60,880	15
COAD	609	4426	3418	313,505	3260	837	132	64,888	37
DLBC	204	1737	111	312,971	1237	456	23	24,229	14
ESCA	229	3219	1279	425,518	1488	506	82	39,625	35
GBM	284	2408	2014	233,660	2475	652	-	56,218	38
HNSC	1245	4793	2041	315,837	3613	811	177	68,380	30
KICH	792	381	2338	69,018	1887	373	45	21,682	9
KIRC	1448	12,270	5669	352,018	7357	4313	124	101,036	108
KIRP	308	2967	4035	322,517	3384	695	96	94,855	47
LAML	485	1485	388	81,027	2093	865	64	11,144	-
LGG	2236	13,263	5853	330,179	9464	3320	289	216,273	129
LIHC	381	3245	3124	310,434	4339	931	145	76,330	25
LUAD	423	4193	2865	279,921	2954	1064	118	43,241	31
LUSC	1258	5732	2323	262,803	3007	955	67	69,637	35
MESO	405	4764	2815	223,561	4818	1395	163	65,016	36
OV	2390	4962	2605	229,452	3521	1202	144	-	55
PAAD	652	5649	4551	253,392	3948	1716	122	49,248	55
PCPG	444	3350	498	167,727	2661	1001	80	68,031	21
PRAD	799	7474	7803	414,995	5706	1958	132	112,158	42
READ	-	3690	1750	136,352	3054	1401	42	24,539	15
SARC	436	3934	8563	295,650	3525	909	181	84,955	85
SKCM	1089	2721	1412	147,333	5691	1244	168	102,533	76
STAD	939	4856	1374	326,289	3218	1145	100	78,419	50
TGCT	139	1677	1138	91,526	801	200	34	5478	7
THCA	39	6168	645	289,537	4914	2387	234	105,462	49
THYM	114	1429	1133	194,424	1208	503	78	35,266	15
UCEC	775	5633	20,415	311,908	6093	1665	149	128,332	80
UCS	148	1955	1067	131,542	1415	808	68	30,502	19
UVM	437	5602	2003	138,783	5202	1324	211	104,545	-

## Data Availability

SurvDB is freely available to the public without registration or login requirements at (https://gong_lab.hzau.edu.cn/SurvDB/, accessed on 1 January 2025).
